# Luciferase Expressing Preclinical Model Systems Representing the Different Molecular Subtypes of Colorectal Cancer

**DOI:** 10.3390/cancers15164122

**Published:** 2023-08-16

**Authors:** Arne Rotermund, Martin S. Staege, Sarah Brandt, Jana Luetzkendorf, Henrike Lucas, Lutz P. Mueller, Thomas Mueller

**Affiliations:** 1 Department of Internal Medicine IV, Hematology and Oncology, Medical Faculty, Martin Luther University Halle-Wittenberg, 06120 Halle, Germany; arne.rotermund@student.uni-halle.de (A.R.); sarah.brandt@uk-halle.de (S.B.); jana.luetzkendorf@uk-halle.de (J.L.); lutz.mueller@uk-halle.de (L.P.M.); 2 Department of Surgical and Conservative Pediatrics and Adolescent Medicine, Medical Faculty, Martin Luther University Halle-Wittenberg, 06120 Halle, Germany; martin.staege@medizin.uni-halle.de; 3 Institute of Pharmacy, Martin Luther University Halle-Wittenberg, 06120 Halle, Germany; henrike.lucas@pharmazie.uni-halle.de

**Keywords:** colorectal cancer, preclinical models, bioluminescence imaging, cell lines, spheroids, xenograft tumors, CMS classification

## Abstract

**Simple Summary:**

More insight into the biological diversity of colorectal cancer (CRC) is needed to improve therapeutic outcomes. We aimed at establishing a combined 2D/3D, in vitro/in vivo model system representing the heterogeneity of CRC with regards to the molecular subtypes, allowing bioluminescence imaging-assisted analyses. Comparative characterization of stable luciferase expressing derivatives of well-established CRC cell lines, derived spheroids and subcutaneous xenograft tumors showed that regarding primary tumor characteristics, the 3D-spheroid cultures resembled xenografts more closely than 2D-cultured cells do. Xenograft tumor growth resulted in metastatic spread to the lungs. Furthermore, a bioluminescence-based spheroid cytotoxicity assay was set up in order to be able to perform dose–response relationship studies in analogy to typical monolayer assays. Thus, the model systems can be used in preclinical research applications to study new therapy approaches and represents the biological heterogeneity of CRC.

**Abstract:**

Colorectal cancer (CRC) is a heterogeneous disease. More insight into the biological diversity of CRC is needed to improve therapeutic outcomes. Established CRC cell lines are frequently used and were shown to be representative models of the main subtypes of CRC at the genomic and transcriptomic level. In the present work, we established stable, luciferase expressing derivatives from 10 well-established CRC cell lines, generated spheroids and subcutaneous xenograft tumors in nude mice, and performed comparative characterization of these model systems. Transcriptomic analyses revealed the close relation of cell lines with their derived spheroids and xenograft tumors. The preclinical model systems clustered with patient tumor samples when compared to normal tissue thereby confirming that cell-line-based tumor models retain specific characteristics of primary tumors. Xenografts showed different differentiation patterns and bioluminescence imaging revealed metastatic spread to the lungs. In addition, the models were classified according to the CMS classification system, with further sub-classification according to the recently identified two intrinsic epithelial tumor cell states of CRC, iCMS2 and iCMS3. The combined data showed that regarding primary tumor characteristics, 3D-spheroid cultures resemble xenografts more closely than 2D-cultured cells do. Furthermore, we set up a bioluminescence-based spheroid cytotoxicity assay in order to be able to perform dose–response relationship studies in analogy to typical monolayer assays. Applying the established assay, we studied the efficacy of oxaliplatin. Seven of the ten used cell lines showed a significant reduction in the response to oxaliplatin in the 3D-spheroid model compared to the 2D-monolayer model. Therapy studies in selected xenograft models confirmed the response or lack of response to oxaliplatin treatment. Analyses of differentially expressed genes in these models identified CAV1 as a possible marker of oxaliplatin resistance. In conclusion, we established a combined 2D/3D, in vitro/in vivo model system representing the heterogeneity of CRC, which can be used in preclinical research applications.

## 1. Introduction

Cancer cell lines are valuable in vitro model systems that are widely used in basic cancer research and drug discovery [1]. Here, the Cancer Cell Line Encyclopedia has clearly demonstrated that large, annotated cell-line collections may help to enable preclinical stratification patterns for anticancer agents [2,3,4]. This has been demonstrated again by two recent studies, which investigated important issues of cancer research such as tumor heterogeneity and metastasis [5,6]. Using cell lines, Jin et al. created a first-generation metastasis map (MetMap) that reveals organ-specific patterns of metastasis associated with clinical and genomic features, and demonstrated the utility of this MetMap [5]. In the second study, Kinker et al. described the landscape of heterogeneity within diverse cancer cell lines and identified recurrent patterns of heterogeneity that are shared between tumors and specific cell lines [6]. Thus, cancer cell lines can be useful models with clinical relevance. 

Colorectal cancer (CRC) is among the most common cancers and a major cause of cancer mortality [7,8]. CRC is a heterogeneous, clinically diverse disease and is poorly understood biologically. Therefore, more insight into the biological diversity of CRC, especially in relation to its clinical behavior, is needed to improve the therapeutic outcomes. Established CRC cell lines are frequently used and are capable of representing the main subtypes of primary tumors at the genomic level, which validates their utility as tools to investigate colorectal cancer biology and drug responses [9]. Furthermore, several groups have characterized primary CRC tumors based on their gene expression data combined with their genomic features in order to establish biologically distinct molecular subtypes with clinical relevance, which resulted in the emergence of different classification systems [10,11,12,13,14,15]. Again, CRC cell lines turned out to reflect the differential subtypes among these classification systems and were capable of being used to test specific targeted therapy approaches [16]. Later, the international CRC Subtyping Consortium (CRCSC) was formed with the aim of resolving inconsistencies among the original reported gene expression-based CRC classification systems. This resulted in the establishment of the CMS classification system consisting of four consensus molecular subtypes (CMS), each with distinguishing properties: CMS1 (microsatellite instability immune, 14%), hypermutated, microsatellite unstable and strong immune activation; CMS2 (canonical, 37%), epithelial, marked WNT and MYC signaling activation; CMS3 (metabolic, 13%), epithelial and evident metabolic dysregulation; and CMS4 (mesenchymal, 23%), prominent transforming growth factor-beta activation, stromal invasion and angiogenesis [17]. In addition, there was a fifth group comprising samples with mixed features, labeled as an intermediate group (13%). Besides the biological differences, clear clinical distinctions were evident between the aforementioned groups. Among others, patients with CMS4 tumors displayed worse overall, and relapse-free, survival. The CMS1 population had very poor survival in the situation after relapse, whereas CMS2 patients showed superior survival after relapse [17]. Furthermore, various studies have also reported on different responses to chemotherapy in the different molecular subgroups of the CMS classification. 

Gene expression profiles of tumor tissue samples represent the sum of signals derived from the cancer cells and their surrounding tumor microenvironment, the latter of which can impede gene expression analysis, depending on the model used. Moreover, the stromal component in a tumor has been suggested to be crucial for the determination of CMS4 [18,19]. In contrast to these reports, Linnekamp et al. characterized a panel of CRC cell culture models including CRC cell lines, primary cultures and PDX models, and clearly detected CMS4 in all model systems, indicating that CMS4 can be defined as a tumor cell-intrinsic phenotype in addition to the observed accumulation of stromal cells [20]. Moreover, Eide et al. developed a novel CMS classifier, referred to as CMScaller, based on cancer cell-intrinsic and subtype-enriched gene expression markers, thereby providing a solution to the analogous problem of classifying pre-clinical models, which either lack a tumor microenvironment entirely (e.g., cell lines and organoid cultures) or present with a completely different background (e.g., murine xenografts) [21,22]. This is particularly relevant for the classification of CMS1 and CMS4, as interactions between tumor cells and microenvironment play an especially important role in these two subgroups. The CMScaller was shown to perform in primary tumors models and recapitulated the biology of the CMS groups, revealing subtype-dependent drug response profiles when applied to PDX and cell lines [23,24]. Moreover, in a recent study, Joanito et al. performed combined single-cell and bulk transcriptome sequencing and identified two intrinsic epithelial tumor cell states in colorectal tumors, which refined the CMS classification system of colorectal cancer [25].

In the present work, we generated stable, luciferase expressing cell clones from established CRC cell lines, in order to analyze growth, therapy response and metastasis in derived spheroid and nude mouse xenograft models utilizing the endogenous signal. Tumor spheroids are useful in vitro models as they can represent several important aspects of real tumor tissues, e.g., (1) three-dimensional growth with structural organization and physiologically relevant cell–cell and cell–matrix interactions; (2) establishment of tumor microenvironmental characteristics such as nutrient gradients, hypoxia and acidosis; (3) localization-dependent heterogeneous cell growth and differentiation; (4) drug resistance mechanisms [26,27]. Mouse xenograft tumors represent aspects of real tumor tissue even more strongly, since they contain a complete stromal component including vasculature and fibrotic tissue, even though being of murine origin. In addition, a residual immune system consisting of B cells, NK cells, macrophages and dendritic cells is still present in these models, especially in athymic nude mice. In this work, we compared the labeled and cloned CRC cell lines with their derived spheroids and xenograft tumors in terms of gene expression and response to therapy. The aim of the study was to establish a useful preclinical model system, which represents the heterogeneity of CRC, especially in regard to the different subtypes according to the CMS classification system. 

## 2. Materials and Methods

### 2.1. Cell Lines and Generation of Luciferase Expressing Clones

The colorectal cancer cell lines HT29 (HTB-38), DLD1 (CCL-221), LOVO (CCL-229), SW48 (CCL-231), LS1034 (CRL-2158), SW1463 (CCL-234), COLO205 (CCL-222), LS174T (CL-188), HCT116 (CCL-247) and SW480 (CCL-228) were originally obtained from the ATCC and were authenticated in 2010 at the DSMZ-German Collection of Microorganisms and Cell Cultures GmbH (Braunschweig, Germany). All cell lines were cultivated in RPMI medium (Sigma-Aldrich, Taufkirchen, Germany) containing 10% fetal bovine serum (BioWest, Nuaillé, France) and 1% penicillin/streptomycin (Sigma-Aldrich) at 37 °C/5% CO_2_ in a humid atmosphere. 

The cDNA of the red-shifted firefly luciferase PLR1 [28], kindly provided by Bruce R. Branchini (Department of Chemistry, Connecticut College, New London, CT, 06320, USA), was cloned into the lentiviral vector system that we previously used [29]. Preparation of the lentiviral particles and the transduction of the used cell lines were performed as previously described [29]. Transduced cell lines were seeded onto 96-well plates to generate single cell clones by means of using limited dilutions. Luciferase (Luc) expressing clones were identified by measuring bioluminescence after supplementation of D-luciferin (Perkin Elmer, Rodgau, Germany) on a Tecan Spark microplate reader (Tecan, Männedorf, Switzerland). Six to ten Luc-positive clones per cell line were picked, expanded and analyzed regarding morphology, growth behavior and drug response in direct comparison to the wild-type cell lines. One clone of each cell line was selected for subsequent experiments. Finally, the newly generated Luc-expressing, cloned cell lines were re-authenticated at the DSMZ in 2020/2021. 

### 2.2. Spheroid Preparation, Growth Kinetics, Drug Treatment 

In order to generate single tumor spheroids, tumor cells resuspended in culture medium were seeded onto 96-well plates, which were coated with 0.7% agarose (SeaKem^®^ GTG™ Agarose, Lonza, Basel, Switzerland) before. The cell lines HT29-Luc, DLD1-Luc, LS174T-Luc, LS1034-Luc and SW1463-Luc were able to form compact spheroids within 2 days. For the cell lines LOVO-Luc, SW48-Luc, COLO205-Luc, HCT116-Luc and SW480-Luc, the used culture medium was supplemented with 10 µg/mL collagen I (Ibidi GmbH, Gräfelfing, Germany) in order to support spheroid formation. Compact spheroids were formed within 7 days. 

In order to analyze spheroid growth, different amounts of cells (range 150–20,000) were seeded onto agarose-coated 96-well plates. The starting point of the assay (d0) was chosen after the formation of spheroids within two or seven days. On d0, 20 µL D-luciferin solution was added per well and luciferase activity was measured after 15 min incubation time on the Spark microplate reader. Further measurements were performed on d2, d5 and d7. Growth kinetic curves were established by plotting mean values (8 spheroids) over time using GraphPad Prism8. Based on these analyses, a cell line-specific cell amount to be used in our cytotoxicity assays was determined: HT29, DLD1, LS174T, LS1034, SW1463–1500; HCT116, LOVO–300; SW48, SW480–500; COLO205–150.

For the cytotoxicity assay, cells were seeded onto agarose-coated 96-well plates and were treated with serial dilutions of oxaliplatin (Eloxatin, 5 mg/mL, provided from own hospital pharmacy) for seven days after compact spheroids formed. Measurements of luciferase activity were performed as described above. Dose–response curves and calculation of IC_50_ values including standard deviations were carried out using GraphPad Prism8. The IC_50_ values of the groups were then compared using a Welch test.

### 2.3. RNA Preparation, Microarray Analysis, Molecular Subtyping and Classification

Cell lines were harvested 48 h after seeding and different samples per cell line were pooled. Spheroids were harvested on day 7 (HT29, DLD-1, LS174T, LS1034, SW1463) or day 12 (LOVO, SW48, COLO205, HCT116, SW480) and were pooled. Xenograft tumors (see Section 2.4) were resected from nude mice when they had reached a volume of approximately 1 cm^3^. Tumors were sliced in half and, afterwards, one half was fixed in formalin for histological analyses, whereas the other half was immediately processed, performing cell separation using the MACS cell separation technology from Miltenyi (Miltenyi Biotech, Bergisch Gladbach, Germany). Preparation of the tumor mass was performed on a gentleMACS Octo by using the Tumor Dissociation Kit (130-095-929), followed by separation using the Mouse Cell Depletion Kit (130-104-694) and the Death Cell Removal Kit (130-090-101) according to the protocols of the manufacturer. For histological analyses, formalin-fixed samples were embedded in paraffin and then cut to perform hematoxylin/eosin (HE) staining according to standard protocols, as described previously [30].

RNA was extracted using the TRIzol™ Reagent (Thermo Fisher Scientific, Waltham, MA, USA) according to the manufacturer’s protocol. For transcriptomic analyses, the Clariom™ D Assay from Applied Biosystems™ (Thermo Fisher Scientific) was used. The processing of the microarrays was performed in the core facility “analyses” of the Center for Medical Basis Research (ZMG) of the Medical Faculty (Martin Luther University Halle-Wittenberg) according to the instructions of the manufacturer. Microarray data will be available from the Gene Expression Omnibus (GEO) database. In addition to our preclinical samples, ten tumor samples and ten normal colon tissue samples from the GEO database (accession number GSE115261 [31]), which was processed using the same microarray, were included in the transcriptomic analyses. 

Microarrays were analyzed using the robust multi-array average (RMA) algorithm with the Transcriptome Analysis Console (TAC4.0; Thermo Fisher Scientific). Gene filtering and cluster analysis was performed with TAC4.0. For the genes included in the cluster analysis focusing on cell line-specific genes and differences between tumor and normal samples were filtered for a false discovery rate (FDR) F-test < 0.0001 and a tumor-versus-normal log2 fold change of >5/<−5 (749 probe sets passed these filters). Normal samples were set as the baseline. Genes included in cluster analysis focusing on the different preclinical model systems were filtered for a FDR F-test < 0.01 and 2D cultures were used as baseline. Genes were further filtered for spheroid-versus-2D and xenograft-versus-2D log2 fold changes of >1.5/<−1.5 (152 probe sets) or >2/<−2 (51 probe sets).

The gene expression data were used to classify the samples according to the CMS classification system applying the CMScaller [21]. The classification was performed according to the instructions provided on https://github.com/peterawe/CMScaller (lastly accessed on 10 August 2023). In addition, the datasets were classified using the CRCassigner [14] according to the instructions provided on https://github.com/syspremed/CRCAssigner (lastly accessed on 10 August 2023).

For classification of the intrinsic epithelial tumor cell type, the specific iCMS2 and iCMS3 gene sets provided in the original study [25] were used to calculate scores for iCMS2. Probe sets were mean collapsed to gene names and for all genes, the sample-specific quantile ranks were calculated. Thereafter, two different scores were calculated: For score A, the means of the quantile ranks for the gene sets [25] iCMS2_up (Mq2U), iCMS2_down (Mq2D), iCMS3_up (Mq3U), and iCMS3_down (Mq3D) were calculated for all samples. Score A was calculated as A = (Mq2U/Mq2D) − (Mq3U/Mq3D). In score B, the sums of the quantile ranks for the same gene sets iCMS2_up (Sqi2U), iCMS2_down (Sqi2D), iCMS3_up (Sq3U) and iCMS3_down (Sq3D) were calculated for all samples. The score B was calculated as B = (Sq2U–Sq2D) − (Sq3U–Sq3D). In both scores, higher positive values suggest that the corresponding sample likely belongs to the iCSM2 class. In addition, nearest template prediction [32] was performed using the mentioned gene sets. Based on the calculated distances to the gene classes iCMS2_up (D2U), iCMS2_down (D2D), iCMS3_up (D3U) and iCMS3_down (D3D), the iCMS classes were determined as: (D2U < D2D) and (D3U > D3U) → iCMS2; (D2U > D2D) and (D3U < D3U) → iCMS3; all other constellations → unstable.

Additional datasets from public databases were used for iCMS classification of additional cell lines. From ArrayExpress, the cel files from dataset E-MTAB-2971 were downloaded. In addition, the following the GEO datasets were used: GSM1374426, GSM1374451, GSM1374452, GSM1374456, GSM1374463, GSM1374516, GSM1374517, GSM1374518, GSM1374561, GSM1374562, GSM1374563, GSM1374564, GSM1374627, GSM1374628, GSM1374629, GSM1374630, GSM1374632, GSM1374633, GSM1374759, GSM1374919, GSM1374920, GSM1374925, GSM1374926, GSM1374927, GSM1374928, GSM1374929, GSM1374930, GSM1374933, GSM1374934, GSM1374935, GSM1374937, GSM206450, GSM206455, GSM206459, GSM206463, GSM206467, GSM206501, GSM206517, GSM206519, GSM206524, GSM206547, GSM206548, GSM206552, GSM206553, GSM206554, GSM843481, GSM843482, GSM844580, GSM844713, GSM887141, GSM887274, GSM887277, GSM887278, GSM887303, GSM887479, GSM887632, GSM887644, GSM887667, GSM887668, GSM887674, GSM887675, GSM887677, GSM887679 (GSE57083, GSE8332 [33], GSE34211 [34], GSE36133 [2]). 

### 2.4. Animal Studies, Treatment, Imaging

Generation of subcutaneous xenograft tumors was performed by inoculation of 5 million tumor cells into the right flank of male athymic nude mice (Charles River Laboratories, Sulzfeld, Germany). Monitoring of tumor growth was performed by caliper measurement and volume calculation using the formula a^2^ × b × π/6 with ‘a’ being the short and ‘b’ the long diameter. For molecular and histological analyses (see Section 2.3), three to four tumors from each cell line model were removed from mice when they had reached a volume of approximately 1 cm^3^. To analyze the ability to metastasize, one tumor of each model was allowed to grow to a size of about 2 cm^3^. After completing, the lungs were removed, incubated in D-luciferin solution for 10 min and bioluminescence imaging was performed on an IVIS Spectrum (PerkinElmer, Rodgau, Germany).

Selected models were used to analyze the anti-tumor activity of oxaliplatin. For this purpose, mice were divided into two groups (*n* = 3) with similar mean tumor volumes of about 0.150 cm^3^ and equal volume distribution at the start of treatment. Treatments comprised weekly intraperitoneal applications of oxaliplatin (8 mg/kg BW) and normal saline (control). Mouse weight and behavior were controlled daily during the course of treatment. The impact of treatment was calculated as the increase in tumor volume after treatment relative to the tumor volume at the start of the treatment on day 0 (mean values ± SD). 

## 3. Results

### 3.1. Luciferase-Labelled CRC Cell Line Clones form Subcutaneous Xenograft Tumors with Various Differentiation Characteristics and Metastasize to the Lung

We chose ten well-known CRC cell lines, which already have been used in recent classification studies, to establish luciferase-labelled derivatives. The red-shifted firefly luciferase PLR1 [28] was cloned into the vector system that we previously used [29], which contains no further selection marker, enabling the utilization of typical selection markers in subsequent experiments. Single cell cloning was performed in order to achieve homogenous cell populations in regard to the chromosomal locus of the incorporated vector cassette. From several picked clones of each cell line, one was selected based on the criteria luminescence intensity, as well as the morphology, growth behavior and drug response resembling the wild type of the corresponding cell line. The generated luciferase (Luc)-expressing, cloned CRC cell lines were re-authenticated using STR analysis.

**Figure 1:**
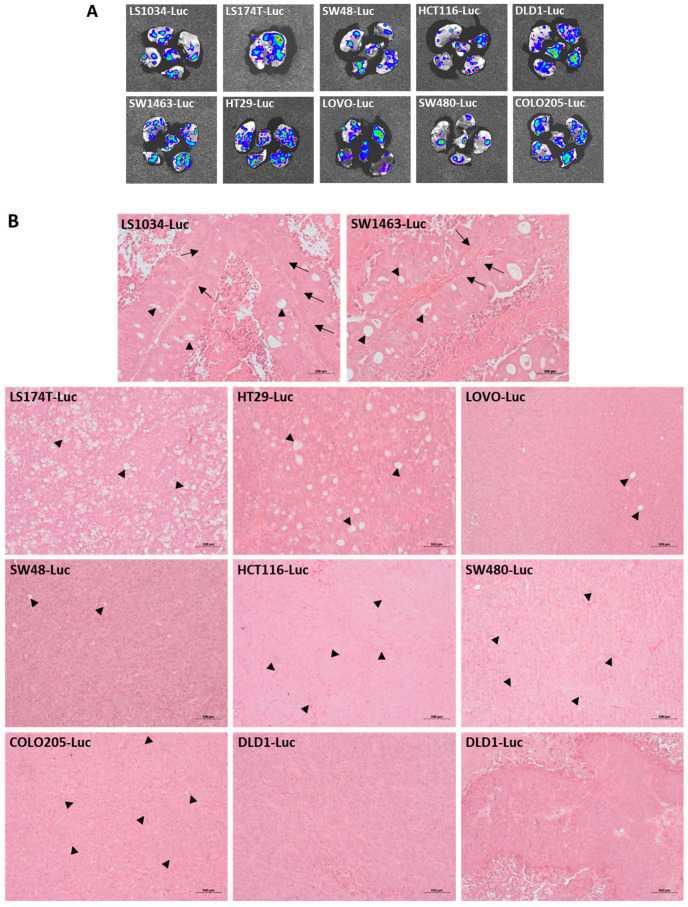
Characteristics of subcutaneous xenograft tumors generated from luciferase expressing CRC cell line clones. (**A**): Lung metastasis analyzed by bioluminescence imaging ex vivo; (**B**): histological examination of tumors after HE staining showing different histological phenotypes. LS1034-Luc and SW1463-Luc: Well differentiated; display of colonic mucosa-like structures with arranged columnar epithelial cells (arrows), as well as goblet cells (arrowheads). LS174T-Luc and HT29-Luc: Occurrence of pronounced goblet cell differentiation (arrowheads). LOVO-Luc and SW48-Luc: Undifferentiated; scattered goblet cells (arrowheads). HCT116-Luc and SW480-Luc: Undifferentiated; high density of small capillary structures (arrowheads). COLO205-Luc: Undifferentiated; high content of mitotic figures (arrowheads). DLD1-Luc: Predominantly undifferentiated (below middle); residual tendency of cellular organization (below right). (Scale bar: 100 µm). For higher resolution display, see original images (Supplementary File S1).

All new Luc-labelled derivatives were able to grow as subcutaneous xenograft tumors in nude mice without any differences to the wild-type cell lines. Interestingly, ex vivo bioluminescence imaging revealed metastatic spread to the lungs in each model (Figure 1A). Histological examination of the subcutaneous tumors showed clear differences (Figure 1B). Tumors from LS1034 and SW1463 cells showed well-differentiated structures resembling those of the colonic mucosa, with arranged columnar epithelial cells including goblet cells. LS174T, and to a lesser extent HT29, tumors were characterized by pronounced goblet cell differentiation and displayed only a residual pattern of layered epithelium formation. Tumors from SW48, LOVO, HCT116, SW480 and COLO205 cells showed a completely undifferentiated phenotype although scattered goblet cells could occasionally be observed. In addition, examination of HCT116 and SW480 tumors revealed a high density of small capillary structures. A high content of mitotic figures was a typical feature of COLO205 tumors, which corresponded with their fast growth compared to the other tumor types. DLD1 tumors predominantly displayed an undifferentiated phenotype but in part showed a residual tendency of cellular organization. Together, these analyses showed that the Luc-labelled, cloned cell lines retained the main properties of wild-type cell lines. 

### 3.2. Luciferase Expressing Cell Lines as Well as Their Derived Spheroids and Xenograft Tumors Represent Molecular Characteristics of CRC

**Figure 2:**
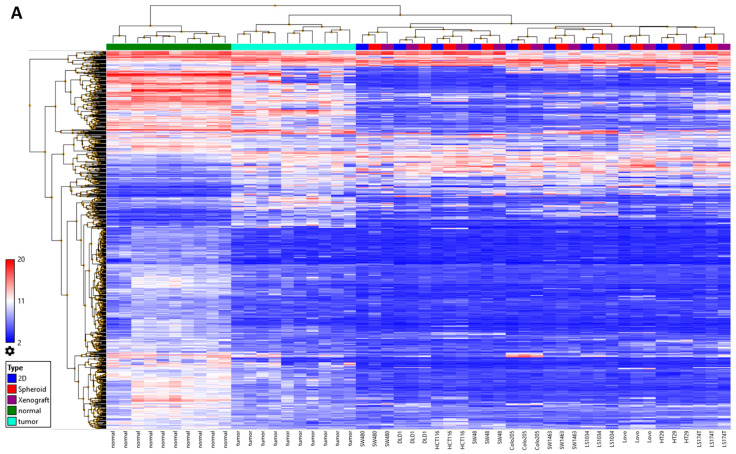

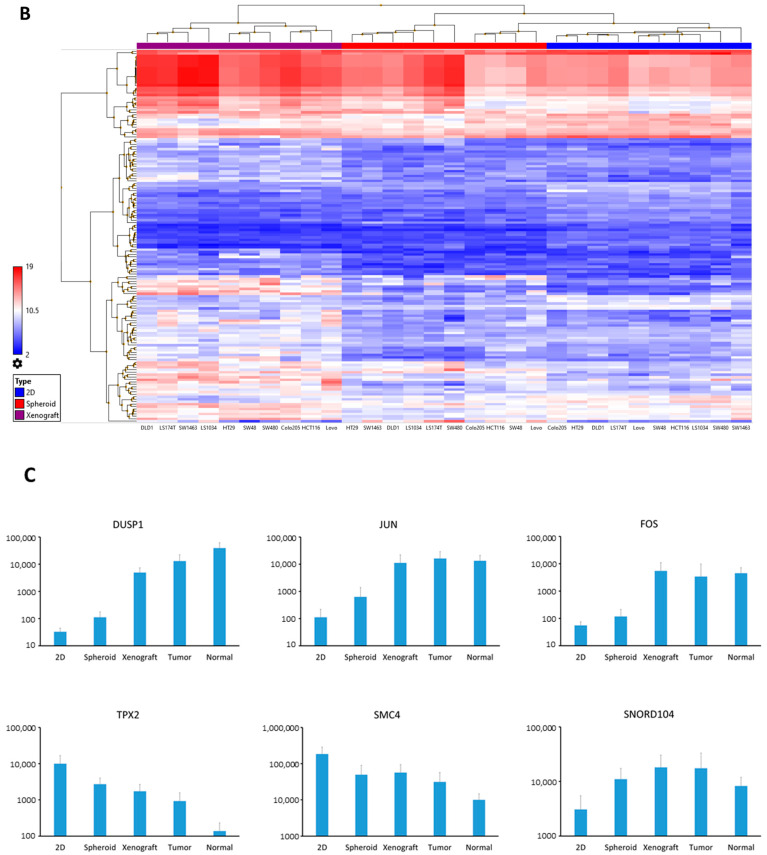
Transcriptomic gene expression and analyses of relationship between cell lines, spheroid and xenograft tumors compared to tumor and normal tissue samples. (**A**): Hierarchical cluster analysis focusing on cell line-specific genes and differences between tumor and normal tissue samples (for parameters see Section 2.3); (**B**): hierarchical cluster analysis focusing on differences among the preclinical model systems (for parameters see Section 2.3); (**C**): examples of genes with model-dependent increasing or decreasing expression pattern compared to tumor and normal tissue samples. Presented are signal intensities. Microarray raw data were analyzed and normalized using the RMA algorithm as indicated in the Materials and Methods section (Section 2.3).

We performed transcriptomic analyses to characterize the Luc-CRC cell lines, derived spheroids and xenograft tumors. The cell lines LS1034, SW1463, HT29, LS174T and DLD1 were able to form compacted spheroids within one or two days. In contrast, SW48, LOVO, HCT116, SW480 and COLO205 only formed loose aggregates in an equal time span. Medium supplementation with collagen I and a longer incubation time was necessary to achieve the formation of properly compacted spheroids in these models. From xenograft tumors, the human tumor cells were extracted and mouse cells were depleted. Furthermore, ten colorectal tumor samples and ten normal colon tissue samples from publicly available data sources [31] were included for further comparison. First, we performed hierarchical cluster analysis focusing on cell line specific genes and differences between the tumor and the normal tissue samples (Figure 2A, gene set 749 in Supplementary Table S1), revealing a close relation between the cell lines and their derived spheroids and xenograft tumors. In addition, clustering the preclinical model systems together with the tumor samples confirmed that the cell line-based tumor models retain specific characteristics of real tumors when compared to normal tissue. Hierarchical cluster analysis focusing on differences among the preclinical model systems showed that spheroids were located in the main cluster together with cell lines when compared with xenograft tumors (Figure 2B, gene set 152 in Supplementary Table S2). Spheroids of the cell lines SW48, LOVO, HCT116 and COLO205 were more closely related to the cell line group than the others. All of these four cell lines belonged to the group, which were less prone to form spheroids. Although belonging to the same group, the cell line SW480 was able to form spheroids faster when supplemented with collagen I, possibly explaining the clustering together with the other group. Furthermore, to investigate the relation between the three preclinical model systems, we performed a correlation analysis (Pearson) based on the gene set 152 (Supplementary Table S2). This revealed that spheroids resemble xenografts more closely than 2D-cultured cells do in each model, with SW48 as the only exception (Supplementary Table S3).

Since spheroid models are described as better reflecting the characteristics of real tumor tissue than cell lines, we questioned whether this phenomenon is associated with a specific pattern of increased or decreased gene expression between the different model systems. Among the 152 genes (Supplementary Table S2) used in the cluster analysis depicted in Figure 2B, 64 genes showed an increasing and 25 genes a decreasing expression pattern. In addition, in three cases, gene expression increased from cell lines to spheroids without further increasing to xenograft tumors, whereas in eighteen cases gene expression decreased without further decreasing in xenograft tumors. Overall, 72% of the 152 genes showed a characteristic pattern indicating that spheroids, more than cell lines, comprise molecular traits of xenograft tumors. Further clustering using a fold-change higher than 2 resulted in a more restricted set of 51 genes, 84% of which showed the described increasing or decreasing expression pattern (Supplementary Table S4). Further analyses in combination with the gene expression data of the tumor and normal tissue samples revealed different, gene-dependent relations between the preclinical models and clinical samples (Supplementary Table S2). For example, the expression of genes such as JUN, FOS and DUSP1 showed an increasing pattern within the model and turned out to be high in both the tumor and normal samples (Figure 2C), suggesting an involvement of these factors in tissue-specific differentiation processes. Decreasing pattern of gene expression within the model associated with lower expression in the tumor and normal samples was found in genes such as TPX2 and SMC4. These genes are involved in cell division processes, thus reflecting the higher proliferation rate of cell lines growing on a monolayer. There were also genes such as SNORD104 whose expression increased from cell lines to tumors, but decreased from tumors to normal tissue, which might point to a tumorigenicity factor. In some cases, gene expression increased within the model, but decreased towards the clinical samples, suggesting the involvement of pure model-specific expression. 

### 3.3. Luciferase Expressing Cell Lines and Their Derived Spheroids and Xenograft Tumors Represent the Main Subtypes of CRC According to the CMS Classification

**Scheme 1:**
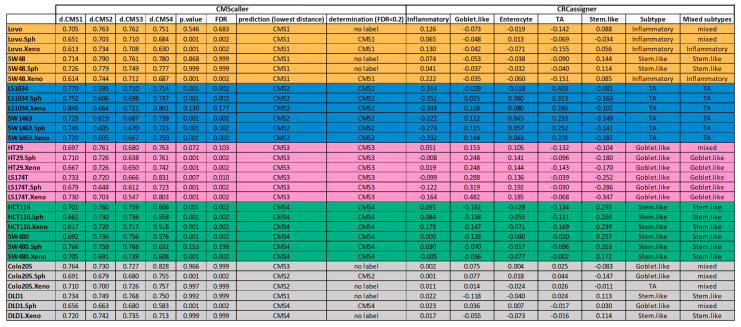
Molecular classification of cell lines, spheroids and xenograft tumors using the CMScaller [21] and the CRCassigner [14].

Next, the CRC models were characterized according to the CMS classification using the CMScaller [21]. In addition, the datasets were analyzed using the CRCassigner, which was developed by Sadanandam et al. as one of the original classification systems, and which uses a cell lineage-related classification [14]. As summarized in Scheme 1, a high concordance between both classification systems could be observed. For example, xenograft tumors of SW48 and LOVO were classified as CMS1 based on the CMScaller and as Inflammatory, which is the corresponding group of the CRCassigner. The concordant CMScaller/CRCassigner classifications of the other xenograft tumors were as follows: LS1034 and SW1463–CMS2/TA (transit amplifying); HT29 and LS174T–CMS3/Goblet-like; HCT116 and SW480–CMS4/Stem-like. Xenograft tumors of COLO205 could not clearly be classified (FDR > 0.2), although there was a clear tendency (lowest distance) to CMS2/TA. DLD1 xenograft tumors were clearly classified as Stem-like by the CRCassigner, which correlated with the closest proximity to CMS4 (lowest distance) obtained by the CMScaller. Interestingly, the clear classification into CMS2/TA and CMS3/Goblet-like correlated well with the specific epithelial differentiation patterns observed in the corresponding xenograft tumors (see Figure 1B). This suggests that the cloned tumor cells still harbor the respective differentiation programs of their origin, which will lead to induction of differentiation processes once they are able to grow as three-dimensional tissue. Moreover, even in those tumors displaying an undifferentiated phenotype, residual signs of cellular differentiation or organization can occasionally be observed.

Comparing the classification of the xenograft tumors with their corresponding spheroids and cell lines, a consistent classification was observed in each model of CMS2 (LS1034, SW1463), CMS3 (HT29, LS174T) and CMS4 (HCT116, SW480) (Scheme 1). Both cell lines of CMS1 (SW48, LOVO) could not be clearly classified (FDR > 0.2), but they showed the closest proximity to the CMS of the respective xenograft tumor. Notably, in the case of LOVO, the corresponding spheroid was clearly classified into CMS1. Spheroids of COLO205 and DLD1 were assigned to CMS2 and CMS4, respectively, in accordance to the characteristic of their respective xenograft tumors, but their respective cell lines showed the closest proximity to a different CMS, i.e., CMS3 for COLO205 and CMS1 for DLD1. This suggest that both models represent samples of the mixed/intermediate group, which is also supported by the data obtained with the CRCassigner (Scheme 1). Taken together, the established panel of Luc-labelled, cloned cell lines represents the heterogeneity of CRC and their derived spheroids and xenograft tumors are capable of clearly recapitulating the main subtypes of CRC according to the CMS classification. 

**Figure 3:**
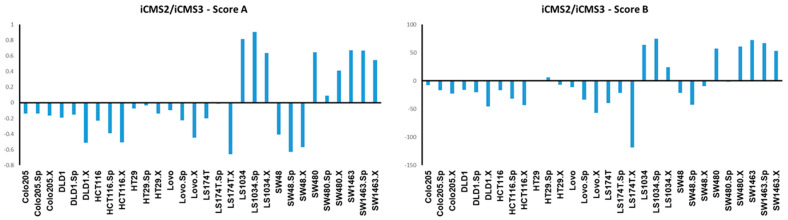
Determination of the intrinsic epithelial subtype iCMS2 vs. iCMS3. (values > 0 means iCMS2 assignment, values < 0 means iCMS3 assignment). Sp: spheroid; X: xenograft tumor.

**Scheme 2:**
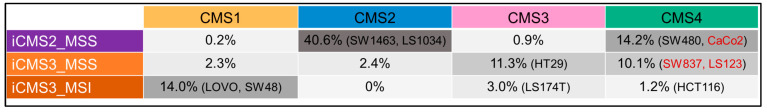
Assignment of the cell models applying combined CMS/iCMS classification. The data of the table are from the original study by Joanito et al. [25] showing the percent distribution of subtypes among colorectal cancer, and were complemented with the model names. Red marked cell lines are in preparation for completion of the CRC model.

In a very recent study, two intrinsic epithelial tumor cell states, iCMS2 and iCMS3, were identified in colorectal tumors, which can be used to refine the CMS classification system [25]. In this dual system, iCMS3 comprises the microsatellite unstable (MSI-H) tumors and one-third of the microsatellite-stable (MSS) tumors. Interestingly, the iCMS3 MSS tumors were transcriptomically more similar to MSI-H tumors than to the iCMS2 MSS tumors. In addition, iCMS3 cancers compared to iCMS2 showed worse survival after relapse. Notably, poor prognosis CMS4 tumors were shown to contain either iCMS2 or iCMS3 epithelium, the latter being associated with the worst prognosis of all subgroups [25]. Using the specific gene sets characterizing iCMS2 and iCMS3 provided in the original study, we analyzed our models. We applied two different scores (see Section 2.3 for explanation) in order to accomplish the assignment to either of the two epithelial subtypes (Figure 3). Both of the CMS1 models, LOVO and SW48, could be classified into iCMS3, with concordance reached between both scoring approaches. Thus, they belong to the most representative group of CMS1 (Scheme 2). LS1034 and SW1463 were clearly classified into iCMS2, which is also the common epithelial type of CMS2. The CMS3 model HT29 could not be clearly assigned, since results of both scores differed (Figure 3). While it rather tends to iCMS3, it seems to harbor characteristics of both epithelial subtypes or represents an intermediate type. Most tumors of CMS3 are iCMS3 with MSS (Scheme 2). The other CMS3 model LS174T, which is MSI-H, turned out to be iCMS3 and therefore belonged to the second group of CMS3. SW480 and HCT116 were classified as iCMS2 and iCMS3, respectively, the first therefore representing one of the two large groups of CMS4, whereas the latter belongs to a small group of CMS4 with MSI-H (Scheme 2). Both intermediate models COLO205 and DLD1 turned out to be iCMS3 (Figure 3). Thus, these models clearly differ in their molecular profile. DLD1 represents the iCMS3-MSI type and combines characteristics of CMS4 and CMS1, whereas COLO205 belongs to the iCMS3-MSS type and harbors characteristics of CMS2/CMS3 tumors. 

**Figure 4:**
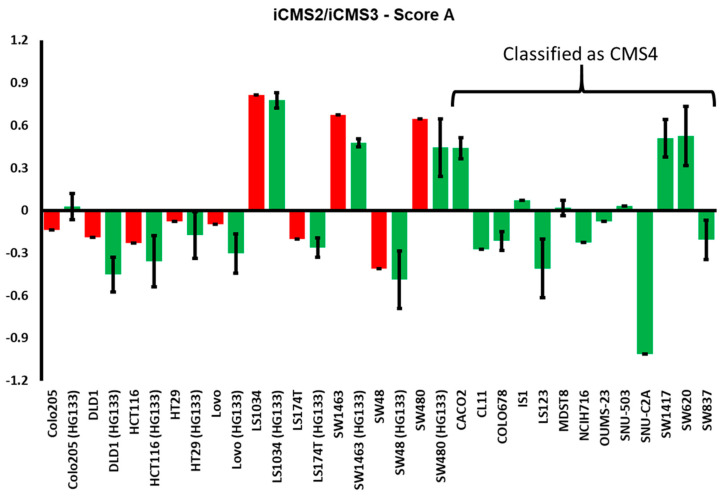
Determination of the intrinsic epithelial subtype iCMS2 vs. iCMS3. (values > 0 means iCMS2 assignment, values < 0 means iCMS3 assignment). Red bars: Cell line models characterized in this study. Green bars: Datasets from public databases (see Section 2.3). Cell lines marked by bracket were classified as CMS4 in a previous study by Sveen et al. [24].

We noticed that neither of our initially selected models represented the second main group of CMS4 tumors CMS4/iCMS3-MSS (Scheme 2). In order to find cell lines with properties of CMS4/iCMS3-MSS, we analyzed a panel of 13 CRC cell lines, which were classified as CMS4 (w/o MSI-H) in a previous study by Sveen et al. [24], using publicly available data sources. In addition, publicly available datasets from the cell lines used in this study were included for comparison. As shown in Figure 4, there was a high concordance between the luciferase expressing models created in this study and the respective datasets from publicly available data sources regarding the iCMS determination, with COLO205 as one exception. HT29 was confirmed to resemble iCMS3 better than iCMS2. Furthermore, from the 13 CMS4 cell lines, 7 could be assigned to the CMS4/iCMS3-MSS type, whereas the others were classified into iCMS2 (Figure 4). Nearest template prediction [32] was performed in addition to the scoring approach to determine the epithelial subtype, which confirmed the stated results (Supplementary Table S5). Based on these data, the well-established cell lines CaCo2, SW837 and LS123 were chosen and will be included into the panel of this study, in order to achieve and guarantee an adequate representation of the main groups of CMS4 to complete the CRC model (Scheme 2). 

Thus, including the three cell lines in preparation, the completed model comprises thirteen CRC cell lines, with four cell lines clearly representing the iCMS2 epithelial subtype (LS1034, SW1463, SW480, CaCo2), whereas seven cell lines (LOVO, SW48, LS174T, HCT116, DLD1, SW837, LS123) clearly belong to the iCMS3 subtype. The cell lines COLO205 and HT29 seem to represent intermediate types with a similarity to iCMS3. Furthermore, Joanito et al. demonstrated that one of the defining features of iCMS2 was the enrichment in copy number variations (CNV), whereas iCMS3 tumors were diploid or showed infrequent and inconsistent CNV [25]. Data regarding CNV of the cell lines could be found in the study of Berg et al., presented as a percent of the genome affected by copy number aberrations [23]. According to these analyses, CNV is consistently high in the iCMS2 cell lines LS1034, SW1463, SW480 and CaCo2 with 42%, 27%, 38% and 47%, respectively, whereas it is low in the iCMS3 cell lines LOVO, SW48, LS174T, HCT116 and DLD1 with 9%, 10%, 9%, 7% and 8%, respectively. Regarding CNV, COLO205 (45%) and HT29 (43%) clearly show features of iCMS2 confirming their nature as intermediate types harboring characteristics of both epithelial subtypes. Thus, the prevalent iCMS2–iCMS3 dichotomy in CRC as well as the occurrence of intermediate types, as reported by Joanito et al., is reproduced in the cell line models. Interestingly, for three out of the seven cell lines representing the specific CMS4/iCMS3-MSS subgroup of CMS4 tumors (Figure 4) data regarding CNV were available in the study of Berg et al. [23], which revealed a rather high CNV, with 18%, 48% and 30% for C11, COLO678 and SW837, respectively. Together, this shows an inconsistent CNV among iCMS3 cell lines, which is in accordance with the inconsistent CNV among the whole group of iCMS3 tumors, as reported by Joanito et al. [25].

### 3.4. Establishing a Bioluminescence Based Cytotoxicity Assay for Spheroid Models

The combined data show that tumor spheroids are more suited to be compared to xenograft tumors in regards to characteristics of patient tumors in comparison with the respective cell lines, making them useful models for preclinical drug research. Taking advantage of the endogenous luciferase expression in these models, we set up a bioluminescence-based cytotoxicity assay in order to be able to perform dose–response relationship studies in analogy to typical monolayer assays. In order to determine the optimal assay conditions, we first studied the growth kinetics of each spheroid model in order to prove the expected correlation between cell amount and signal intensity as an important prerequisite. A direct correlation between spheroid mass and signal intensity was confirmed in freshly formed spheroids. However, a near linear growth kinetic in growing and compacted spheroids is highly dependent on the amount of cells seeded at the start of the experiment. The relative decrease in signal intensity in growing spheroids can be explained by their typical characteristics such as the induction of oxygen and nutrient gradients leading to hypoxia, acidosis and heterogeneous cell growth, with proliferating cells at the rim area, and less proliferating/differentiated or even apoptotic/necrotic cells in the core area [35,36,37]. The characteristic decrease in signal intensity during spheroid growth was observed in each model to a different extent requiring a cell line-specific cell amount to be seeded at the start of the assay (see Section 2.2).

Applying the established assay, we studied the efficacy of oxaliplatin in the spheroid models of the whole panel. Oxaliplatin treatment induced a dose-dependent inhibition of spheroid growth and resulted in a typical dose–response pattern. Next, IC50 values were calculated and compared with existing data obtained from monolayer assays. This revealed clear differences throughout the whole panel (Table 1). In general, seven of the ten used cell lines showed a significantly reduced response to oxaliplatin, and thus a significant increase in IC50 values, when comparing the monolayer model with the spheroid model. This effect is of particular relevance considering that the spheroid assay comprises a prolonged drug treatment of 7 days compared to the monolayer assay (4 days). The alterations in IC50 values also resulted in a different sensitivity pattern within the panel. For example, on the monolayer level, SW1463, LS174T, LOVO and SW48 cells represented the most sensitive cell lines. However, the latter two turned out to be less sensitive in our assay on the spheroid level (LOVO: 0.13 -> 0.37; SW48: 0.08 -> 0.49), whereas the first two remained the most sensitive towards oxaliplatin treatment, indicated by their comparably low IC50 values (Table 1; SW1463: 0.09 -> 0.10; LS174T: 0.11 -> 0.18). The differential gain in resistance cannot be simply explained by the differential spheroid morphology, so that, for example, higher compactness leads to hindered drug penetration. For instance, COLO205 has the greatest increase in IC50 value in the 3D spheroid model, but forms the least compacted spheroids, whereas the spheroids of sensitive SW1463 and LS174T are very dense and compact. This suggests that the differential oxaliplatin sensitivity is instead determined by molecular mechanisms.

### 3.5. Analyzing Response to Oxaliplatin in Nude Mice Xenograft Tumors

**Figure 5:**
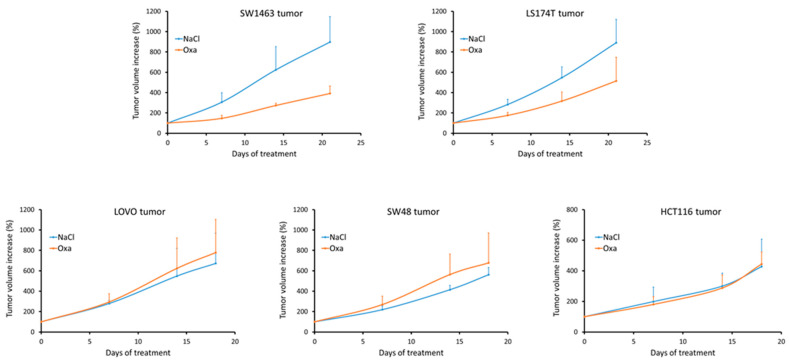
Antitumor activity of oxaliplatin in nude mice xenograft tumors of SW1436, LS174T, LOVO, SW48 and HCT116. Mice were treated with NaCl (control) or oxaliplatin (8 mg/kg BW) on days 0, 7 and 14. The tumor volume increase relative to the start of treatment on day 0 is shown, as mean values ± SD (*n* = 4, LS174T, LOVO, HCT116; *n* = 3, SW1463, SW48).

We next questioned to what extent the different in vitro sensitivity of spheroids, represented by their specific IC50 value, can predict a specific response towards oxaliplatin treatment in vivo. We therefore analyzed the impact of an oxaliplatin therapy in selected xenograft models (Figure 5). Clear response to oxaliplatin resulting in a substantial tumor growth inhibition over time could only be observed in the SW1463 model. A reduced yet still moderate overall tumor growth inhibition was achieved in the LS174T model. Tumors of the models LOVO, SW48 and HCT116 were completely resistant to oxaliplatin treatment. These results confirmed the lack of oxaliplatin sensitivity of LOVO and SW48 models, which was assumed based on the analyses in the spheroid model. Together, the presented findings suggest that IC50 values above 0.2 µM in the spheroid assay may predict a lack of response to oxaliplatin treatment in the xenograft model. The only selective activity of oxaliplatin within the model reflects the low overall therapeutic activity of this drug as a single agent in CRC. 

### 3.6. Identification of CAV1 as a Putative Marker of Oxaliplatin Resistance

Based on the proven oxaliplatin resistance, we performed analyses of differentially expressed genes comparing the xenograft tumors of SW1463 and LS174T with those of LOVO, SW48 and HCT116 in order to find targets associated with the differential oxaliplatin sensitivity. CAV1 (caveolin 1) was identified as the top-ranked gene with a 400-fold increased expression in resistant vs. sensitive xenograft tumors, (Supplementary Table S6). Furthermore, CAV1 turned out to be the top-ranked gene (375-fold) when performing the same analyses using the spheroid models. In the 2D models, CAV1 was the second listed gene with a 280-fold increased expression in resistant cells. This suggests that a possible CAV1-associated mechanism of oxaliplatin resistance is based on cell intrinsic characteristics in one part, but is further supported under 3D growth conditions. Interestingly, CAV1 has already been linked with drug resistance in general [39] as well as specifically in CRC [40,41,42]. Therefore, further analyses to explore the role of CAV1 in CRC especially with regard to oxaliplatin-containing chemotherapy are worth performing. 

## 4. Discussion

Cancer is among the world’s greatest health problems and one of the leading causes of death worldwide [7]. Since cancer itself is a very heterogeneous and still often poorly understood disease, both from a clinical and a biological perspective, more research is necessary to improve the prevention and treatment of this deadly disease. Cancer cell lines are important and useful tools used especially in preclinical cancer research and drug discovery due to their availability and comparability amongst others [1]. Due to the heterogeneity of cancer and many new discoveries in the area of targeted medicine, the emphasis on treatment stratification is as high as ever before. Therefore, preclinical research needs to establish models, which are able to reproduce the distinct molecular patterns of in vivo tumors in vitro based on standardized cancer cell lines. Promising research was conducted on this topic in recent years, showing the potential of cancer cell line models [5,6]. 

As CRC is both a leading cause of cancer mortality as well as a very diverse and poorly understood disease, establishing an aforementioned model is of utmost importance, in order to improve preclinical research with the aim of improving therapeutic options. Previous research has already suggested that CRC cell lines have the potential to be used as representative models [9]. The CMS classification established a consistent CRC classification system, dividing CRC into several subgroups with different patterns and properties [17]. Several studies have demonstrated significant differences concerning outcome and efficacy of chemo- and targeted drug therapy connected to the different molecular subtypes [24,43,44,45,46,47,48]. Interestingly, the molecular stratification of CRC is far from finished as recent work has described the existence of two intrinsic epithelial tumor cell states, iCMS2 and iCMS3, in colorectal tumors, therefore further refining the CMS classification [25]. The so-called IMF-classification was introduced, which is based on the discovered dichotomy of malignant epithelial cells, as well as the microsatellite status and the occurrence of fibrosis inside the tumor tissue [25]. 

Our panel of luciferase-labeled, cloned CRC cell lines was able to robustly recapitulate the main subtypes of CRC based on the CMS classification in the majority of cases, both in the spheroid as well as in the mouse xenograft model and to lesser extent in the monolayer model. The results for the cell lines were in accordance to the results obtained for the wild-type cell lines in previous reports [23,24]. Further characterization of the models applying combined CMS/iCMS classification including MSS/MSI status revealed the lack of representation of the specific CMS4/iCMS3-MSS subgroup of CMS4 tumors, requiring the complementation with further cell lines. Thus, including the three cell lines in preparation, the completed model comprises thirteen CRC cell lines, which represent the main subtypes of the confined CMS classification integrating the iCMS2–iCMS3 dichotomy (Scheme 2), but also different intermediate types. Therefore, the heterogeneity of CRC is reproduced in the cell line-derived models. 

The combined data showed that 3D-spheroid cultures resemble xenografts more closely than 2D-cultured cells do regarding primary tumor characteristics, which can be expected. While unrestrained proliferation is the main task for cancer cells in monolayer models, three-dimensional growth now requires cell–cell interactions and the establishment of a tumor microarchitecture [49]. This notion is supported by our finding that two genes, TPX2 and SMC4, both essential proteins involved in mitosis and cell division, are among the genes which are downregulated in the xenograft and spheroid model, when compared to the 2D monolayer model, whereas JUN, FOS and DUSP1, genes with a role in differentiation processes, were among the most upregulated genes [50,51]. JUN and FOS are part of the transcription factor AP-1, which plays an important role in cell growth and the differentiation and overexpression of these two proteins leads to an increased expression of other oncogenes in several cancer entities [52,53,54]. DUSP1 on the other hand plays a role in carcinogenesis, tumor progression and response to anti-cancer treatment, as expression of DUSP1 is essential for the resistance of lung cancer cell lines to cisplatin treatment [55,56]. In addition, small nucleolar RNA SNORD104 was identified as a marker whose expression increased from cell lines to tumors, but decreased from tumors to normal tissue, which might point to a tumorigenicity factor in CRC. Interestingly, in a recent report, the overexpression of SNORD104 was shown to promote endometrial cancer growth in preclinical models in vivo and in vitro [57]. Furthermore, SNORD104 was among a marker panel identified as a novel snoRNA expression signature associated with overall survival in patients with lung adenocarcinoma [58]. Therefore, further investigation to explore the role of SNORD104 in CRC is worth performing.

Tumor spheroids are useful in vitro models and are also an easier, cheaper and faster way to create a three-dimensional preclinical model compared to mouse xenograft tumors [26,27]. Recent studies conducted on spheroids also showed the ability of this three-dimensional model to improve preclinical research in the fields of drug discovery, drug penetration, tumor metabolism and tumor migration, among others [59,60,61,62]. Furthermore, recent research by Koch et al. showed differences in the chemo- and radioresistance of four established CRC cell lines between a two-dimensional monolayer and a three-dimensional spheroid model [63]. This is in accordance with our observation regarding the different sensitivity to oxaliplatin. Thus, the establishment of a three-dimensional structure and the associated mechanisms may play a role in the development of chemotherapeutic resistance, a common problem for CRC patients, as CRC is capable of a vast number of mechanisms to achieve chemotherapeutic resistance leading to a worse outcome for patients [64]. However, the spheroid model, of course, has limitations when compared to an in vivo tumor, as the first grows in artificial medium and completely lacks the possibility of an interaction between tumor cells and cells of the tumor stroma or the immune system. Mouse xenograft tumor models contain stromal components and a residual immune system, albeit of murine origin, is present even in athymic mice making it an even more realistic clinical model. Nevertheless, our in vitro spheroid model was able to predict a lack of response to oxaliplatin treatment in vivo. CAV1 was identified as a possible marker for oxaliplatin resistance based on analyses in xenograft tumors, which could be completely reproduced in the spheroid models further confirming the usefulness of the in vitro 3D model.

Analysis of the differential oxaliplatin sensitivity with respect to the molecular subtypes represented by the models revealed some interesting clues. For example, a subset of patients with CMS2 featured tumors benefited from oxaliplatin containing chemotherapy regimen compared to other subtypes in the adjuvant setting [48]. Accordingly, only one of our two CMS2 models (SW1463) turned out to be sensitive to oxaliplatin treatment. Furthermore, meta analyses of results of further clinical studies, summarized in Ten Hoorn et al. [46], revealed an overall inferior effect of oxaliplatin-containing therapy compared to irinotecan-based therapy in all other groups than CMS2 in the metastatic setting, with a clear superior effect of the latter in CMS4 tumors. Regarding the iCMS2-iCMS3 dichotomy, a difference in sensitivity to the single drugs, 5-fluorouracil, SN38 and oxaliplatin, in representative models was found to be not significant [25]. Accordingly, among our two oxaliplatin-sensitive models, one was iCMS2 (SW1463) and one iCMS3 (LS174T). In addition, Joanito et al. evaluated two sets of genes whose expression was correlated with drug response [25]. Interestingly, gene sets positively correlated with drug sensitivity to FOLFOX regimen were upregulated in iCMS2 cells and genes correlated with drug resistance were downregulated, whereas iCMS3 cells showed patterns of up- and downregulation suggesting responsiveness to FOLFIRI [25]. This is in accordance with the observations mentioned above, since almost all CMS2 tumors are composed of iCMS2 epithelium, whereas almost all CMS1, almost all CMS3 and half of CMS4 tumors harbor iCMS3 epithelium (see Scheme 2). Together, this confirms the predictive value of specific subtyping of CRC.

## 5. Conclusions

With our work we aimed to establish a combined 2D/3D, in vitro/in vivo model system capable of representing the heterogeneity of CRC with regards to the molecular subtypes, and allowing bioluminescence imaging-assisted analyses. Our work has also shown that spheroid models do exhibit a higher similarity towards xenograft tumors compared to monolayer models both in terms of expression patterns as well as in terms of response to drug treatment due to the establishment of a three-dimensional structure and the associated mechanisms. Although spheroids do not completely resemble the heterogeneity found inside xenograft and human tumor samples, they are very solid models, especially considering their easy manageability and availability in comparison to xenograft models, with a stronger validity when, for example, performing drug screening studies.

## Figures and Tables

**Table 1:** Oxaliplatin specific IC50 values (µM) ± SD obtained from cytotoxicity assays performed in the monolayer- and the spheroid models and analysis of differences. (Monolayer data are from own previous studies [38]). (*: *p* ≤ 0.05, **: *p* ≤ 0.01, ***: *p* ≤ 0.001, ns: not significant).

Cell Line	Mean Monolayer	SD Monolayer	Mean Spheroid	SD Spheroid	*p*-Value	Significance
LOVO	0.13	0.02	0.37	0.09	0.0009	***
SW48	0.08	0.00	0.49	0.12	0.0005	***
LS1034	0.30	0.05	0.37	0.14	0.3537	ns
SW1463	0.09	0.04	0.10	0.03	0.7680	ns
HT29	0.26	0.04	2.12	0.97	0.0053	**
LS174T	0.11	0.04	0.18	0.04	0.0778	ns
HCT116	0.24	0.01	1.39	0.33	0.0003	***
SW480	0.43	0.12	2.40	0.95	0.0035	**
COLO205	0.27	0.12	12.44	6.77	0.0159	*
DLD1	2.02	0.36	4.10	0.84	0.0012	**

## Data Availability

The datasets used and/or analyzed in the current study are available from the corresponding author upon reasonable request.

## Supplementary Materials

The following supporting information can be downloaded at: https://www.mdpi.com/article/10.3390/cancers15164122/s1, Supplementary File S1: HE stains-original images; Supplementary Table S1: cluster cell line plus tumors vs. normal_749 genes; Supplementary Table S2: model_152 genes_with tumors and normal; Supplementary Table S3: Pearson correlation_2D-Sph-Xeno; Supplementary Table S4: model_51 genes_with tumors and normal; Supplementary Table S5: iCMS determination by NTP; Supplementary Table S6: DEG analyses_Oxa res. vs. sens._2D-Sph-Xeno.
