# Supplementary figures and images for: Luciferase Expressing Preclinical Model Systems Representing the Different Molecular Subtypes of Colorectal Cancer
Source: Cancers (Basel). 2023 Aug 16;15(16):4122. doi: 10.3390/cancers15164122 (PMC10452405; doi:10.3390/cancers15164122)

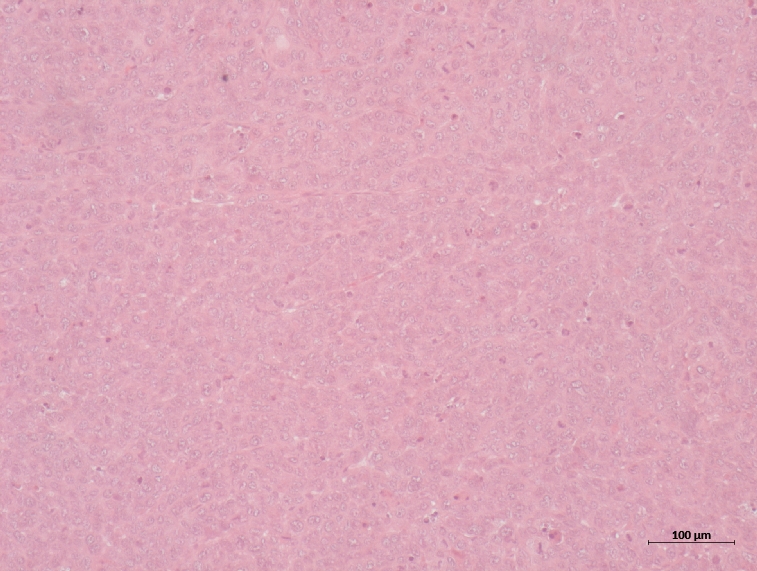

Supplement: Supplementary file 1 [file cancers-15-04122-s001.zip › Suppl. File S1_HE stains-original images/COLO205.jpg]

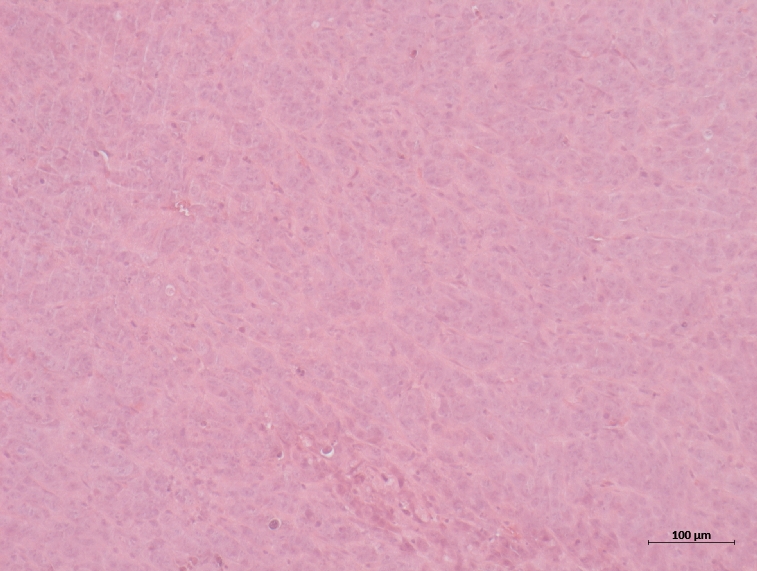

Supplement: Supplementary file 1 [file cancers-15-04122-s001.zip › Suppl. File S1_HE stains-original images/DLD1 1.jpg]

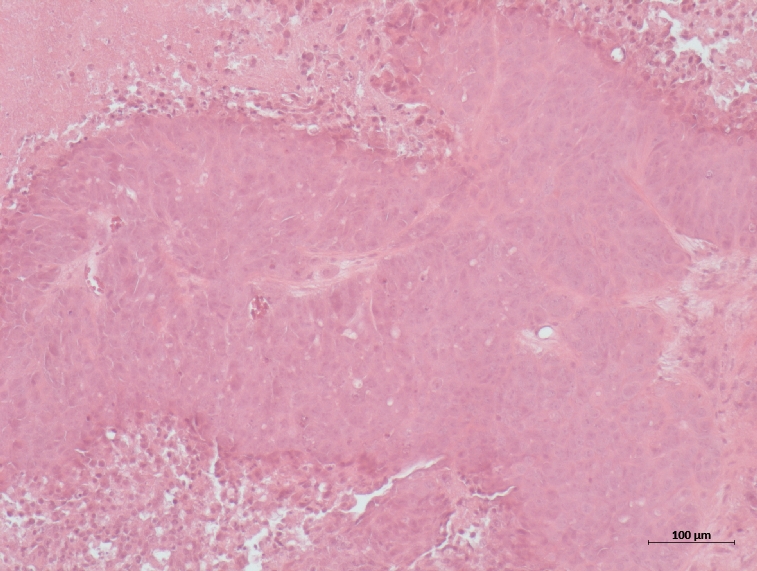

Supplement: Supplementary file 1 [file cancers-15-04122-s001.zip › Suppl. File S1_HE stains-original images/DLD1 2.jpg]

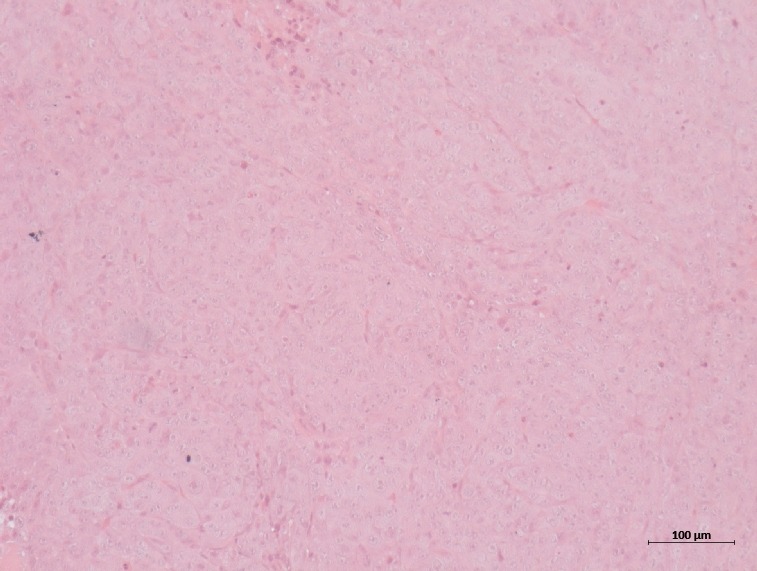

Supplement: Supplementary file 1 [file cancers-15-04122-s001.zip › Suppl. File S1_HE stains-original images/HCT116.jpg]

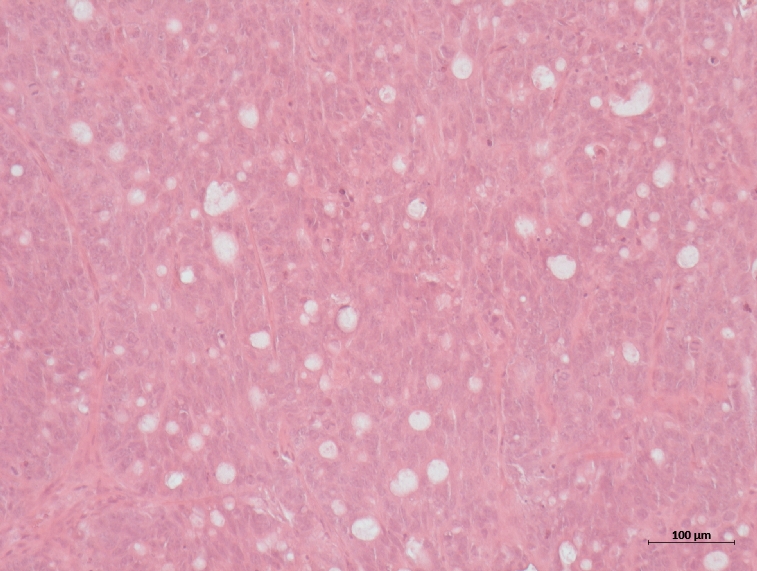

Supplement: Supplementary file 1 [file cancers-15-04122-s001.zip › Suppl. File S1_HE stains-original images/HT29.jpg]

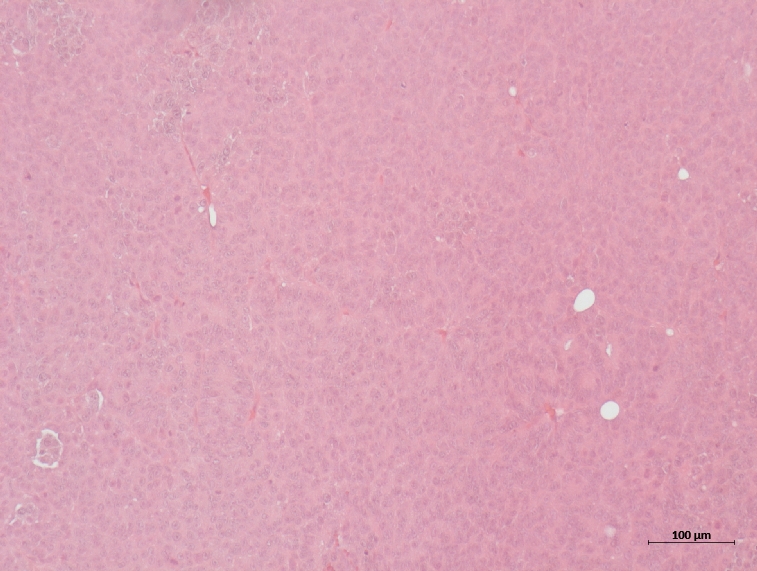

Supplement: Supplementary file 1 [file cancers-15-04122-s001.zip › Suppl. File S1_HE stains-original images/LOVO.jpg]

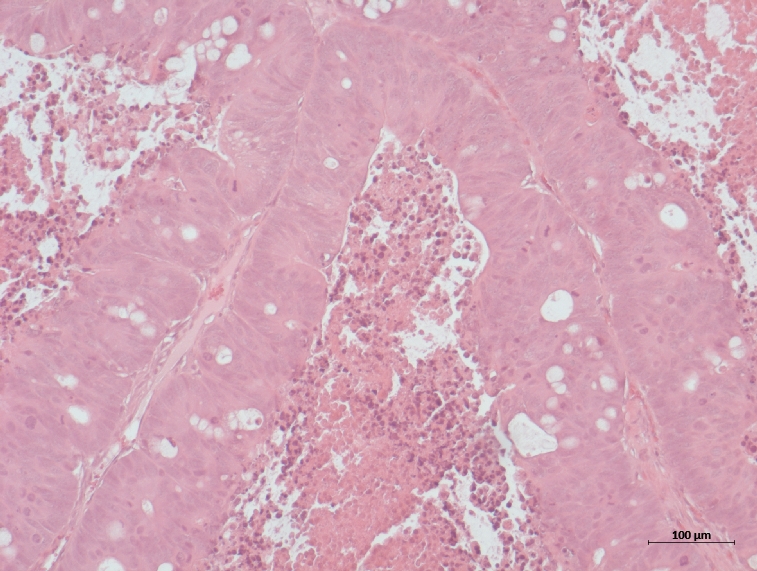

Supplement: Supplementary file 1 [file cancers-15-04122-s001.zip › Suppl. File S1_HE stains-original images/LS1034.jpg]

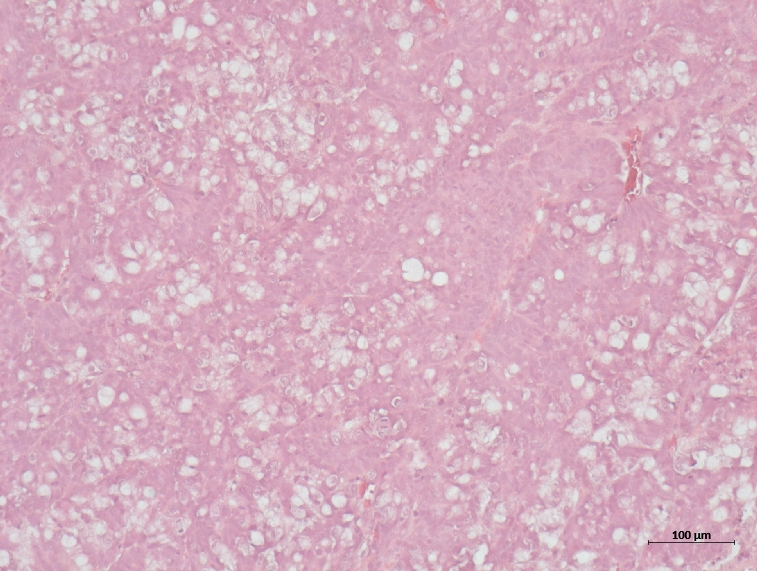

Supplement: Supplementary file 1 [file cancers-15-04122-s001.zip › Suppl. File S1_HE stains-original images/LS174T.jpg]

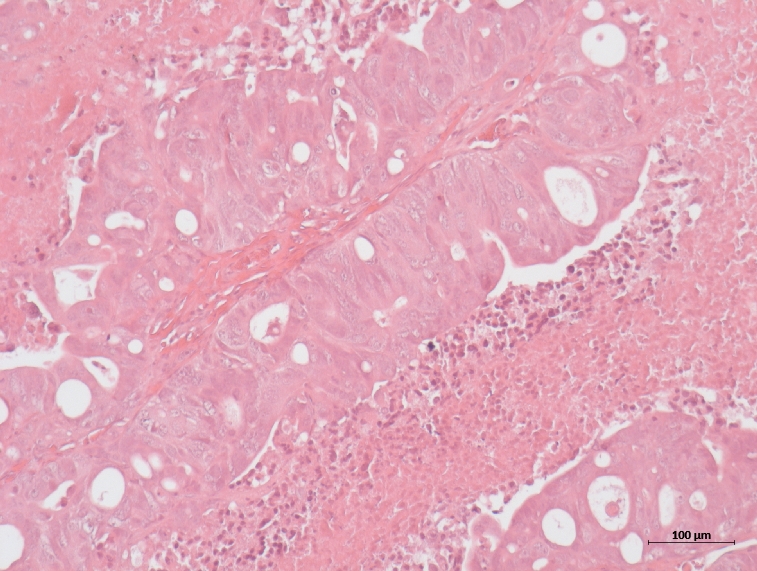

Supplement: Supplementary file 1 [file cancers-15-04122-s001.zip › Suppl. File S1_HE stains-original images/SW1463.jpg]

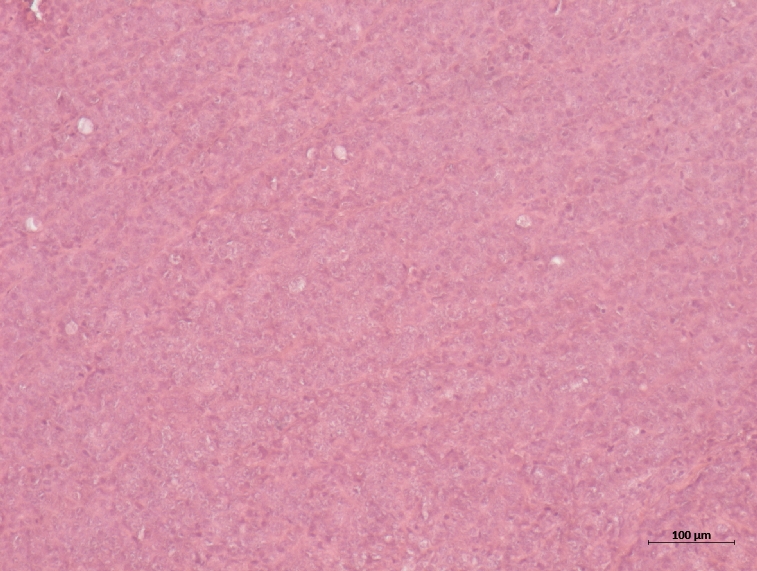

Supplement: Supplementary file 1 [file cancers-15-04122-s001.zip › Suppl. File S1_HE stains-original images/SW48.jpg]

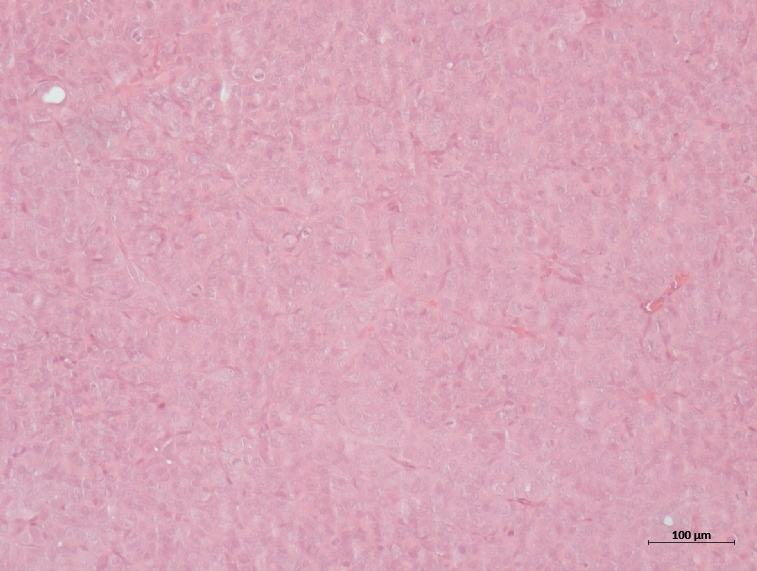

Supplement: Supplementary file 1 [file cancers-15-04122-s001.zip › Suppl. File S1_HE stains-original images/SW480.jpg]
